# Bangpungtongsung-san for patients with major depressive disorder: study protocol for a randomized controlled phase II clinical trial

**DOI:** 10.1186/s12906-023-03912-1

**Published:** 2023-04-12

**Authors:** Yunna Kim, Yujin Choi, Mi Young Lee, Seung-Hun Cho, In Chul Jung, Dong-Hoon Kang, Changsop Yang

**Affiliations:** 1 Department of Neuropsychiatry, College of Korean Medicine, Kyung Hee University Medical Center, Kyung Hee University, Seoul, Republic of Korea; 2 Research Group of Neuroscience, East-West Medical Research Institute, WHO Collaborating Center, Kyung Hee University, Seoul, Republic of Korea; 3 KM Science Research Division, Korea Institute of Oriental Medicine, Daejeon, Republic of Korea; 4 KM Convergence Research Division, Korea Institute of Oriental Medicine, Daejeon, Republic of Korea; 5 Department of Oriental Neuropsychiatry, College of Korean Medicine, Daejeon University, Daejeon, Republic of Korea

**Keywords:** Major depressive disorder, Phase II study, Bangpungtongseong-san, Fangfengtongsheng-san, Bofu-tsusho-san, Herbal medicine, Clinical protocol, Randomized controlled trial

## Abstract

**Background:**

Bangpungtongsung-san (BTS) is a representative herbal medicine that has been widely used for patients with obesity in east Asian countries. Various preclinical studies have demonstrated the anti-depressive effect of BTS granules in various animal models of depression. This phase II trial aimed to explore the efficacy and safety of BTS in human patients with depression.

**Methods:**

A total of 126 patients diagnosed with major depressive disorder and who are not underweight (body mass index ≥ 18.5 kg/m^2^) will be enrolled in this study. Eligible participants will be randomly allocated into three groups: the high-dose BTS, low-dose BTS, and placebo groups in a 1:1:1 ratio. BTS or placebo granules will be orally administered twice a day for 8 weeks. The BTS and placebo granules will be made to have identical color, scent, and shape, and participants and investigators will be blinded to the allocation. The primary efficacy endpoint is the change from baseline of the 17-item Hamilton Depression Rating Scale total score at 8 weeks. The superiority of the high- and low-dose BTS granules to the placebo granules will be tested.

**Discussion:**

The results of this clinical trial will provide evidence on the efficacy and safety of BTS for patients with major depressive disorder. This study will be conducted in accordance with ethical and regulatory guidelines, and the results will be submitted and published in international peer-reviewed journals.

**Trial registration:**

CRIS registration Number: KCT0007571; registered on 2022/07/26 (https://cris.nih.go.kr/cris/search/detailSearch.do/23192).

**Supplementary Information:**

The online version contains supplementary material available at 10.1186/s12906-023-03912-1.

## Background

Major depressive disorder (MDD) is a common mental disorder whose estimated global prevalence after the coronavirus disease-2019 pandemic is 3,153 cases per 100,000 population [1]. One of the most critical problems of MDD is its repeated relapse and recurrence, with a recurrence rate of 50% and 85% in 6 months and 10 years, respectively [2]. Most patients with MDD experience repetitive episodes and undergo chronic progress. The etiology of MDD comprises severe social problems, such as suicide, which is the fifth leading cause of death in Korea [3], or a negative perception of the disease, which results in a passive approach to receiving treatment [4]. Another problem is the high discontinuation rate of antidepressant treatment; 43.5% of patients with MDD discontinue treatment at 6 weeks [5]. Accordingly, a new drug for depression with few side effects and a low risk of drug dependence is necessary.

Patients with MDD share a concomitance of depressed mood and lethargy. However, symptoms of changes in sleep and appetite occur differently among patients with MDD [6]. These symptoms can be divided into melancholic depression, atypical depression, anxious depression, and a mix of the aforementioned manifestations [7]. Atypical depression is characterized by mood reactivity and at least two of the following symptoms: increased appetite or weight gain, hypersomnia, leaden paralysis, and interpersonal rejection sensitivity [8]. In literature, atypical depression is defined according to two symptoms–increased appetite/weight gain and hypersomnia [9]. In the UK Biobank Mental Health Survey, atypical depression showed earlier onset, more recurrent episodes, and higher severity. Patients with atypical depression had higher rates of comorbid obesity, cardiovascular disease, and metabolic syndrome [9]. According to a meta-analysis of anthropometric studies on subtypes of depression, the atypical depression group showed 2.55 times higher body mass index (BMI) than the typical depression group [10]. These epidemiological traits imply that an approach different than that for typical depression should be used for atypical depression, considering its comorbidity and progress. As one of the major differences in mechanism between atypical and melancholic depression is increased inflammation shown by increased levels of proinflammatory cytokines and C-reactive protein [11], a novel medicine should be suggested for patients with atypical depression.

Bangpungtongseong-san (BTS) is one of the most used formulas in traditional east Asian medicine and has been widely sold as an over-the-counter drug for weight control in Korea [12]. In Korea, about 40 products containing BTS extract have been approved as over-the-counter drugs by the Ministry of Food and Drug Safety. The approved indications for BTS extract are accompanying symptoms of hypertension (palpitations, stiff shoulders, and flushing), obesity, swelling, and constipation. The novel efficacies of BTS other than those described in traditional medical bibliographies are being continuously unveiled through clinical and preclinical studies. BTS has been reported to be efficient in various diseases, ranging from metabolic diseases, such as hypertension, lipid abnormalities, and diabetes, to skin diseases, such as herpes zoster, chronic urticaria, and inflammatory dermatitis [13]. Accordingly, BTS is considered to have the potential for its indications to be expanded to other metabolic and inflammatory diseases. Furthermore, the antidepressant and anti-neuroinflammatory effects of BTS extract were found in in vivo and in vitro studies [14].

This clinical trial aimed to test the efficacy of BTS for patients with MDD in a human study. Particularly, considering the characteristics of BTS, which has been widely used for obesity, we plan to include normal-weight or overweight patients with MDD, excluding those who are underweight. Several clinical trials on BTS in obese patients have demonstrated positive results [15–17]. Moreover, we identified that BTS extract is a herbal medicine that shows a high expression of anti-depressant-like effects among the approved herbal medicine products in Korea [14]. Accordingly, we plan to study BTS as an effective alternative for patients with MDD who are over normal weight, have an excessive appetite, and suffer from weight gain.

The main objective of this phase II trial is to find the appropriate dose of BTS granules in patients with MDD for a further confirmative phase III trial. The primary efficacy endpoint is the change from baseline of the 17-item Hamilton Depression Rating Scale (HDRS) total score at 8 weeks. The mean difference of the primary efficacy endpoint will be compared between the high-dose and low-dose BTS groups and the placebo group. If either two doses of BTS show superiority over the placebo, a further confirmative phase III trial will be planned. The secondary efficacy endpoints include the response and remission rate of depression, as defined by the 17-item HDRS total score, and depression severity, as measured by the Beck Depression Inventory-II (BDI-II). Moreover, the safety of administrating BTS granules for 8 weeks compared to that of placebo granules will be evaluated.

## Methods/design

### Trial design and setting

This clinical trial is designed as a randomized, controlled, investigator- and participant-blinded multicenter trial. Three groups will be included: the high-dose BTS, low-dose BTS, and placebo groups. The enrolled participants will be randomly allocated to each group in a 1:1:1 ratio. The superiority of the high- and low-dose BTS granules to placebo granules will be tested. This clinical trial will be conducted in two academic hospitals in the Republic of Korea. This protocol follows the Standard Protocol Items: Recommendations for Interventional Trials (SPIRIT) Statement (see Additional file 1).

### Eligibility criteria

#### Inclusion criteria

This clinical trial will enroll men and women aged 19–65 years diagnosed with MDD according to the Diagnostic and Statistical Manual of Mental Disorders-5 (DSM-5) criteria. The baseline 17-item HDRS total score of enrolled participants should be ≥ 18, and the baseline BMI should be ≥ 18.5 kg/m^2^. Only participants who provide informed consent can be included.

#### Exclusion criteria

The exclusion criteria are as follows: participants at high risk of suicide; requiring hospitalization due to MDD; diagnosed and being treated for panic disorder, obsessive disorder, post-traumatic stress disorder, or personality disorder; having a history of manic, schizophrenic, or mixed episodes; having current or lifetime alcohol or other substance abuse/dependence disorders; having a medical condition that may affect depression severity, such as hypothyroidism or hyperparathyroidism; or having an unstable medical condition, such as uncontrolled hypertension or diabetes, liver dysfunction, or renal impairment. Participants who received nonpharmacological treatments for depression, such as electroconvulsive therapy, vagal nerve stimulation, or deep brain stimulation within 3 months, or those who took medicines that may affect depression severity, such as anxiolytics, antipsychotics, corticosteroids, or hormone replacement therapy within 4 weeks, will be also excluded. To prevent adverse events, participants demonstrating loose stool for more than 3 times a day within 7 days, taking other laxatives, or with symptoms of abdominal pain, vomiting, or loss of appetite due to digestive disorders will be excluded. Moreover, pregnant or lactating women or participants determined to be unsuitable by the investigators will be excluded. The detailed inclusion and exclusion criteria are presented on the clinical trial registration webpage (https://cris.nih.go.kr/cris/search/detailSearch.do/23192).

### Interventions

#### BTS and placebo granules

Three BTS or placebo granule sachets (1 g for each sachet; total, 3 g) will be orally administered twice a day for 8 weeks. Participants in the high-dose BTS group will take three BTS granule sachets, those in the low-dose BTS group will take one BTS and two placebo granule sachets, and those in the placebo group will take three placebo granule sachets for one dosage. The BTS granule was approved by the Ministry of Food and Drug Safety (product code: 197,900,572). One gram of the BTS granule sachet contains 0.5 g of soft-extract BTS as an active ingredient. Soft-extract BTS is prepared by decocting the 18 herbs presented in Table 1 together with 8 to 10 times the amount of water boiling at approximately 80–100 °C for 2–3 h. After vacuum concentration under 60 °C, approximately 3.0 g of soft-extract BTS was obtained. The aforementioned amount, which will be taken on the daily be the high-dose BTS group, contains at least 3.8 mg of *Glycyrrhizic acid*, 2.7 mg of *Paeoniflorin*, 0.6 mg of total alkaloid (*Ephedrine* and *Pseudoephedrine*), 15.4 mg of *Baicalin*, and 5.4 mg of *Geniposide*. Three-dimensional chromatogram of BTS sample based on High-performance liquid chromatography-photodiode array analysis can be found in the previous report [14]. The placebo granule does not contain any active ingredients and has been developed to have an identical appearance (lemon yellow granule) and scent to those of the BTS granule. The BTS and placebo granules are manufactured and packaged by Hanpoong pharmaceuticals (Jeonju, Republic of Korea) according to the good manufacturing practice guideline for medicinal products.

**Table 1:** Name and dosages of herbal substances for 3 g of soft-extract BTS

Herbal name	Botanical name	Part used	Dosage (g)
Angelica gigas	Angelica gigas Nakai [Apiaceae]	Root	0.60
Paeonia lactiflora	Paeonia lactiflora Pall. [Paeoniaceae]	Root	0.60
Cnidium officinale	Ligusticum officinale (Makino) Kitag. [Apiaceae]	Rhizome	0.60
Forsythia viridissima	Forsythia viridissima Lindl. [Oleaceae]	Fruit	0.60
Mentha arvensis	Mentha arvensis L. [Lamiaceae]	Shoot	0.60
Saposhnikovia divaricata	Saposhnikovia divaricata (Turcz.) Schischk. [Apiaceae]	Root	0.60
Ephedra sinica	Ephedra sinica Stapf [Ephedraceae]	Stem	0.60
Rheum undulatum	Rheum undulatum L. [Polygonaceae]	Root and rhizome	0.75
Natrii sulfas	-	-	0.75
Platycodon grandiflorum	Platycodon grandiflorus (Jacq.) A.DC. [Campanulaceae]	Root	1.01
Scutellaria baicalensis	Scutellaria baicalensis Georgi [Lamiaceae]	Root	1.01
Gypsum	-	-	1.50
Zingiber officinale	Zingiber officinale Roscoe [Zingiberaceae]	Rhizoma	0.60
Gardenia jasminoides	Gardenia jasminoides J. Ellis [Rubiaceae]	Fruit	0.60
Schizonepeta tenuifolia	Nepeta tenuifolia Benth. [Lamiaceae]	Flower stalk	0.60
Atractylodes japonica	Atractylodes lancea (Thunb.) DC. [Asteraceae]	Rhizome	1.01
Glycyrrhiza uralensis	Glycyrrhiza uralensis Fisch. ex DC. [Fabaceae]	Root and rhizome	1.01
Talcum	-	-	2.51

#### Criteria for discontinuing allocated interventions

If a serious adverse event occurs or a participant wants to discontinue administration owing to an adverse event, the administration of the investigational product will be stopped. Moreover, in case depression becomes too severe or the risk of suicide has become high in a participant during the trial, the investigators are to determine whether administration should be discontinued. The risk of suicide will be closely assessed using the Columbia Suicide Severity Rating Scale [18, 19] at every visit. In case of discontinuing allocated interventions, the safety assessment will be conducted as planned, if possible.

#### Procedure for monitoring adherence

The compliance rate of administering investigational products, defined as the percentage of the number of BTS or placebo granule sachets actually taken according to the number of sachets that should be taken, will be checked at every visit by pharmacists. The number of sachets actually taken will be checked by the number of empty sachets returned from the participant. Participants will be educated on how to take the BTS or placebo granules by pharmacists at every visit. Participants whose compliance rate is ≥ 75% will be included in the per-protocol set.

#### Permitted and prohibited concomitant interventions

The medications taken 4 weeks before participating in the trial and not among the following prohibited medications can be permitted as concomitant medications; antidepressants, anxiolytics, antipsychotics, corticosteroids, and hormone replacement therapy are prohibited. Medications or herbal medicine that can affect depressive symptoms, as well as non-pharmacological interventions for improving depression, including acupuncture, meditation, electrical stimulation, and magnetic stimulation, are also prohibited. Moreover, bulk-forming and osmotic laxatives are prohibited. Meanwhile, medications administered for the purpose of the transient treatment of diseases other than depression can be permitted following assessment by investigators.

### Outcomes

#### Primary outcome

The primary objective is to evaluate the effect of BTS on depressive symptoms compared to that of the placebo granules. The difference between the two treatment arms (high-dose vs. placebo and low-dose vs. placebo) in the change from baseline of the 17-item HDRS total score at 8 weeks is the primary endpoint of this trial [20].

#### Secondary outcomes

Secondary objectives include evaluating the effect of BTS compared to that of the placebo granules on depressive symptoms with clinician-rating and self-rating outcomes at different time points. The change from baseline of the 17-item HDRS total score at 2, 4, 6, and 12 weeks, as well as the response and remission rates defined by the 17-item HDRS total score at 8 weeks and 12 weeks, will be assessed as secondary outcomes. The response rate will be defined as the percentage of participants in each group whose 17-item HDRS total score improved by more than 50%. The remission rate will be defined as the percentage of participants in each group whose 17-item HDRS total score is under 7 points [21]. Secondary outcomes also include the change in the participants’ self-rated BDI-II total score from baseline to at 4, 8, and 12 weeks [22].

The secondary objectives also include evaluating the effect of BTS on anxiety, anger, insomnia, and quality of life compared to that of the placebo granules. State and trait of anxiety will be assessed using State-Trait Anxiety Inventory (STAI) [23]. Moreover, state anger, trait anger, and anger expression will be assessed using the State-Trait Anger Expression Inventory (STAXI) [24]. The severity of insomnia will be assessed by Insomnia Severity Index (ISI) [25], and the quality of life will be assessed using the 3-level version of the EuroQol-5 Dimension (EQ-5D-3L) index [26]. These outcomes will be measured at baseline, and at 4, 8, and 12 weeks.

#### Exploratory outcomes

**Fig. 1:**
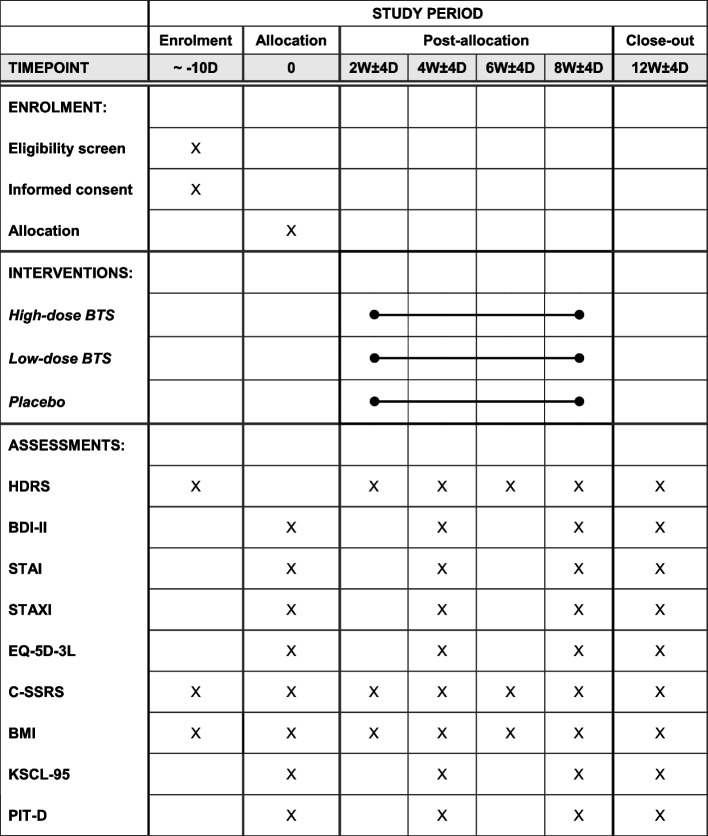
Schedule of enrolment, interventions, and assessments. BTS, Bangpungtongsung-san; HDRS, Hamilton Depression Rating Scale; BDI-II, Beck Depression Inventory-II; STAI, State-Trait Anxiety Inventory; STAXI, State-Trait Anger Expression Inventory; EQ-5D-3L, 3-level version of the EuroQol-5 Dimension; C-SSRS, BMI, Body Mass Index; KSCL-95, Korean Symptom Check List 95; and PIT-D, Pattern Identifications Tool for Depression

The exploratory objectives of this trial include exploring predictive factors for the treatment response. The height and weight of each participant will be measured at the screening visit, and weight will be measured every visit to calculate BMI. Moreover, Korean Symptom Check List 95 [27] and Pattern Identifications Tool for Depression [28] will be assessed at baseline, and at 4, 8, and 12 weeks. A schematic diagram of participant timelines is presented in Fig. 1.

### Sample size and recruitment

The total sample size has been estimated to be 126, with 42 participants for each group. To our knowledge, no previous clinical trial has compared the effect of BTS on depressive symptoms with that of placebo granules, and the effect size of BTS was estimated based on the result of a previous clinical trial on another herbal medicine in patients with MDD [29]. The sample size was calculated based on the hypothesis that the mean change from baseline of the 17-item HDRS total score in the high-dose BTS group is higher to that in placebo group. The mean difference in score between the high-dose BTS and placebo group was estimated to be 4.0 at 8 weeks, and the pooled standard deviation was estimated to be 5.78. With the significance level (α) of 0.05, statistical power (β) of 0.80, allocation ratio of 1:1, and drop-out rate of 0.05, the required sample size for each group was determined to be 42 participants. The participants for this clinical trial will be recruited from two university hospitals in Korea.

The recruitment will be posted on the hospital bulletin boards and online homepage. Moreover, local advertisements on the subway and online advertisements will be conducted to reach the target sample size within the planned period. All recruitment posters and methods have prior institutional review board (IRB) approval.

### Random allocation and blinding

An independent statistician generated a random allocation sequence using SAS® version 9.4 (SAS Institute Inc., Cary, NC, USA). The manager of the random allocation sequence provided the generated random allocation sequence to the pharmaceutical company to pack the investigational product. The high-dose BTS, low-dose BTS, and placebo granules were packed into the allocated random number based on the sequence provided. The investigators will assign a random number to each participant according to the order of enrollment in visit 2. Allocation will be concealed to the investigators by sequential numbering.

The participants, investigators, pharmacist, and outcome assessor will be blinded to the allocated group of each participant. The placebo granule has been developed to have identical color, scent, and taste to those of the BTS granule. In case of a serious medical emergency, unblinding of the group allocation of the participant can be considered. When sub-investigators or principal investigators judge that code-breaking is required, the principal investigator will quickly hold a meeting among investigators and make a decision as to whether to unblind through discussion. A case of unblinding and related medical issues will be reported to the IRB until 24 h.

### Data collection and management

To increase the reliability and validity of the HDRS measured as the primary outcome in this study, investigators in charge of assessing the HDRS at the two sites were trained using a structured interview guide for the HDRS [30]. The validated Korean version of HDRS [31], as well as those for BDI-II [32], STAI [33], STAXI [34], ISI [35], and EQ-5D-5L [36], will be used in this trial. The investigators will check the participants’ understanding and the missing values for all responded questionnaires. The list of measurements that will be used in this clinical trial is presented in Table 2.

**Table 2:** Overview of measurements

Instrument	Measured variable
**Primary outcome**	
HDRS	Clinician-rated depression
**Secondary outcomes**	
BDI-II	Self-rated depression
STAI	Anxiety
STAXI	Anger
ISI	Insomnia
EQ-5D-3L	Quality of life
**Exploratory outcomes**	
BMI	Obesity
KSCL-95	Psychological screening
PIT-D	KM patterns for depression

*HDRS* Hamilton Depression Rating Scale, *BDI-II* Beck Depression Inventory-II, *STAI* State-Trait Anxiety Inventory, *STAXI* State-Trait Anger Expression Inventory, *ISI* Insomnia Severity Index, *EQ-5D-3L* 3-level version of the EuroQol-5 Dimension, *BMI* Body mass index, *KSCL-95* Korean Symptom Check List 95, *PIT-D* Pattern Identifications Tool for Depression, *KM* Korean medicine

The investigators will send regular messages to participants to encourage them to complete the clinical trial. In case a participant discontinues administration or drops out from the trial, the investigators will attempt to have an assessment visit within 1 week for safety and HDRS follow-up.

The data will be entered into a case report form (CRF) on an electronic data capture system. In the system, the ranges for data values were set to avoid the entry of obvious outliers. The clinical research associates will conduct 100% source document verification between the data recorded in the CRF and data in the source documents. The system and manual query will be reviewed monthly after the first participant is enrolled. Comorbidities, medical history, and adverse events will be coded using the MedDRA dictionary, and drug history will be coded using ATC code.

### Statistical methods

Data will be analyzed using SAS® version 9.4 (SAS Institute Inc., Cary, NC, USA). In the efficacy analysis, the full analysis (FA) set will be used as the main analysis set, and the per protocol (PP) set will be used as the supplementary analysis set. The FA set will include randomized participants and minimize the use of those excluded from the analysis. A participant who has never taken the investigational product or has never been evaluated since the random allocation will be excluded from the FA set. The analyses of data containing missing values will be handled with the multiple imputation method. The PP set will include participants who completed the trial without major protocol violations. Participants who drop out during the intervention period (8 weeks), who are found to be inappropriate for inclusion according to the eligibility criteria, or whose total compliance rate is under 75% will be excluded from the PP set. In the safety analysis, the safety set will include all participants who have ever taken the investigational product.

The primary efficacy endpoint is the change from baseline of the 17-item HDRS total score at 8 weeks. The mean difference in outcomes will be compared between the two groups using an analysis of covariance with the site and baseline value as covariates. Tests will be conducted twice to compare the outcomes of the high- and low-dose BTS groups with that of the placebo group. For multiple parallel-group comparisons, a significance level (α) of 0.025 and statistical power (β) of 0.80 will be used for each test. To analyze continuous outcomes among the secondary outcomes (e.g., BDI-II, SRAI, STAXI, ISI, and EQ-5D-3L index scores), an identical analysis method to the primary outcome will be used. To analyze binary outcomes among the secondary outcomes, response to treatment and remission rate of depression will be assessed, and a logistic regression analysis will be conducted with the site and baseline value as covariates. The method to handle multiple comparisons is identical to that used for analyzing continuous outcomes.

Additionally, a subgroup analysis with various criteria will be conducted for exploratory purposes. First, subgroups will be classified based on whether participants are of healthy weight or overweight, as assessed by BMI. Second, other subgroups will be defined using the baseline response to the “changes in appetite” item in BDI-II. Third, some subgroups will be defined using the baseline Korean medicine pattern identification of depression. Moreover, in case of significant differences in the baseline demographic information between groups, adjusted analyses can be conducted.

### Data monitoring and auditing

This clinical trial uses an approved herbal medicine product for another indication. The BTS granules do not have any serious adverse events in the real world, and the risk of this trial is expected to be low. Moreover, it is a phase II trial, meaning that a data monitoring committee is not needed. Interim analyses are not planned.

Adverse events will be carefully collected on every visit after the administration of the investigational product. The severity of adverse events will be rated as mild, moderate, and severe. The causality of adverse events with the investigational product will be categorized as follows: definitely related, probably related, possibly related, probably not related, and definitely not related. Adverse events of dyspepsia, diarrhea, and abdominal pain can occur after the administration of BTS granules, and these symptoms will be carefully checked.

Regular monitoring is planned, with frequent visits planned on being conducted for each 4–5 participants enrolled. After the initiation visit, the first regular monitoring visit will be conducted within 7 working days after the first participant is enrolled. Compliance with the IRB-approved clinical protocol, collection of data, written informed consent of participants, recording and reporting of adverse events, management of the investigational product, data entered into the Electronic Data Capture system, and study materials will be checked in the regular monitoring visits.

### Protocol amendments

Protocol modifications will be determined after sufficient discussion by investigators at the hospital and Korea Institute of Oriental Medicine and will be applied to the study after obtaining approval for the amendment from the IRB. The current version of the protocol is 1.8 (date: 2022–06-27).

### Confidentiality and post-trial care

The personal information of each participant will not be entered into an electronic CRF, and the data of each participant will be collected under screening and random numbers.

Follow-up observation will be conducted at week 12, that is, 4 weeks after the completion of intervention. Compensation criteria and planning regarding those who suffer harm from participation in this trial are prepared. The occurrence of adverse events will be checked at every visit, and required treatment and observation will be applied until the symptoms disappear.

### Dissemination policy

The clinical study information and results will be registered to the Clinical Research Information Service. The findings of this study will be presented at conferences and published in peer-reviewed journals. The participant-level dataset will be uploaded to the Korean Medicine Data Repository (kmdr.kiom.re.kr) after completing the study. Moreover, we will report the final data to the Ministry of Health and Welfare, Republic of Korea, through the Korea Health Industry Development Institute. Results will also be published following completion of the study.

## Discussion

The proposed study will examine the efficacy and safety of BTS administration for 8 weeks among patients with MDD with a BMI ≥ 18.5 kg/m^2^ compared with those of the placebo. We designed the trial to investigate both low-dose and high-dose BTS by allocating the participants into high-dose BTS, low-dose BTS, and placebo groups in a 1:1:1 ratio. In addition to assessing depression using the HDRS total score as the primary outcome, the response and remission rates, anxiety, anger, insomnia, and quality of life will be measured.

The current study will compare both low and high doses of BTS, as low-dose BTS showed better efficacy for depression in animal studies, even though the commonly used dose for obese human patients is that equivalent to the high dose used in this trial. As obesity and depression share biological pathways, BTS is expected to work on both obesity and depression [37]. Obesity causes hypothalamic–pituitary–adrenal dysregulation and changes in the plasma levels of cortisol, leptin, adiponectin, resistin, and insulin, which are hormones involved in emotional and mood regulation [38]. Obesity and depression are vulnerable when an imbalance in appetite and homeostasis dysregulation of the central nervous system occurs. The association between both diseases is complex and bidirectional [37]. These findings suggest that the medications with indications for obesity can be repositioned as new antidepressants as there is a possibility of common mechanisms for both diseases. Thus, it is expected that BTS may be particularly effective for patients with atypical depression, especially those who have bulimia or weight gain. These aspects were not only reflected in inclusion criteria, but also in the additional analyses that were planned, which included subgroup analyses of patients who are overweight based on BMI and of patients who responded that they have increased appetite in the depression symptom evaluation during screening.

Moreover, this clinical trial considered pattern identification [39], an important feature of diagnosis and clinical decision-making in Korean medicine (KM). Herbal medicines for depression are often prescribed according to the individuals’ pattern identification [40]. To design clinical trials on herbal medicine, the disease studied should be defined clearly in both conventional medical and traditional eastern Asian medical approaches [41]. This clinical trial will recruit patients with MDD following the DSM-5 diagnostic approach, and pattern identification will be partially implemented using the objective measurement of BMI. By excluding patients with underweight BMI, we intended to exclude patients who are not adequate for BTS prescription.

This study has some limitations. First, even though we planned to develop and use BTS in patients with atypical depression, we did not adopt the diagnosis of the atypical depression subtype and only excluded patients who are underweight. The feasibility for recruiting each participant in the clinical trial was considered, and we attempted to use objective criteria for recruitment as much as possible. Second, as this is a placebo-controlled phase II trial, comparison of BTS with commonly used antidepressants, such as selective serotonin reuptake inhibitors, is warranted in a future phase III definitive trial.

This phase II trial will provide information on the efficacy and safety of BTS in patients with MDD who are of healthy weight or overweight. The findings of this randomized controlled study are expected to provide evidence for a novel approach to depression with fewer side effects and a low risk of drug dependence, especially in the atypical depression subtype.

## Supplementary Information

**Additional file 1. **SPIRIT checklist.

## Data Availability

Not applicable.

## Abbreviations

BTS: Bangpungtongsung-san
MDD: Major depressive disorder
BMI: Body mass index
HDRS: Hamilton Depression Rating Scale
BDI-II: Beck Depression Inventory-II
SPIRIT: Standard Protocol Items: Recommendations for Interventional Trials
DSM-5: Diagnostic and Statistical Manual of Mental Disorders-5
STAI: State-Trait Anxiety Inventory
STAXI: State-Trait Anger Expression Inventory
ISI: Insomnia Severity Index
EQ-5D-3L: 3-Level version of the EuroQol-5 Dimension
IRB: Institutional review board
CRF: Case report form
FA: Full analysis
PP: Per protocol
